# Effect of SARS-CoV-2 (COVID-19) Pandemic and Lockdown on Body Weight, Maladaptive Eating Habits, Anxiety, and Depression in a Bariatric Surgery Waiting List Cohort

**DOI:** 10.1007/s11695-021-05257-5

**Published:** 2021-02-21

**Authors:** Umberto Albert, Pasquale Losurdo, Alessia Leschiutta, Serena Macchi, Natasa Samardzic, Biagio Casaganda, Nicolò de Manzini, Silvia Palmisano

**Affiliations:** 1 Department of Medicine, Surgery and Health Sciences, University of Trieste, Trieste, Italy; 2 Department of Mental Health, UCO Clinica Psichiatrica, Azienda Sanitaria Universitaria Giuliano-Isontina (ASUGI), Trieste, Italy; 3 Surgical Clinic Unit, Azienda Sanitaria Universitaria Giuliano-Isontina (ASUGI), Trieste, Italy

**Keywords:** Bariatric surgery, SARS-CoV-2, COVID-19, Maladaptive eating habits

## Abstract

**Purpose:**

On January 30, 2020, the World Health Organization declared COVID-19 as a “public health emergency of international concern.” The primary aim of the study was to evaluate weight and food habit changes during COVID-19 outbreak. The secondary endpoint was to explore the psychological factors, arising during the pandemic, influencing weight and dietary variations.

**Materials and Methods:**

A survey composed of four different items was conducted by telephone interview: (1) anthropometric data and type of procedure, (2) Hospital Anxiety and Depression Scale (HADS), (3) maladaptive eating behaviors, and (4) personal feelings moved by the COVID-19 spread and lockdown.

**Results:**

Fifty-six patients were enrolled. No significant changes in weight, BMI, and maladaptive eating habits were observed. A significant reduction in the anxiety index score was observed. In 17.8% of cases, a change in obesity class was reported, and among these patients, a substantial modification in bariatric procedures was planned (60%).

**Conclusion:**

This study showed no effect on weight and BMI nor on rates of maladaptive eating habits associated with quarantine/social isolation among severely obese individuals waiting for the bariatric surgery. At the end of lockdown, a considerable proportion of patients modified their initial obesity class, and in selected cases, it could represent a criteria for rearrangement of the planned bariatric procedure. In obese patients, the lockdown and social distancing generated a reduction of fear of confronting and being negatively judged by others. This psychological aspect was assessed with the reduction of the HADS score.

## Introduction

On January 30, 2020, the World Health Organization (WHO) declared SARS-CoV-2 (COVID-19) infection as a “public health emergency of international concern” [1]. From then on, the pandemic escalated rapidly. Consequently, hospitals and institutions of care handled a rising number of infected patients beyond all expectations, and a deep reorganization of national health systems has become necessary to face the pandemic.

To contain the spread of the virus, we have been witnessing the implementation of strict measures unprecedented in modern times. Major cities and entire nations imposed mass quarantines and “red zones,” recommended to stay at home, and finally imposed lockdown with restrictions on incoming and outcoming travelers and on gatherings as well as closure of schools and businesses [2, 3].

Regarding surgical field, restrictions involved all non-urgent procedures, and as a consequence, all surgeries in elective setting were stopped and bariatric operations were re-scheduled [4–6].

In Italy, the lockdown was declared on March 8, 2020 and the area worst hit by the coronavirus outbreak was the northern part, especially Lombardy, Piedmont, Emilia Romagna, and Veneto regions. Our study was conducted in Friuli Venezia Giulia region that is an area bordering Veneto, so an increased alert and an early implementation of strategies coping with virus spread were put in place. The aggressiveness of the virus in this part of Italy, the seriousness of the outbreak, the scale of contagion, and the number of deaths had a great emotional impact on the population.

Considering that, the development of obesity is multifactorial, and the emotional component represents a key factor [7, 8], it is possible that these elements could have influenced eating behaviors. During the lockdown, the negative emotions felt by general population might have been fear of contagion, boredom, and distress, due to the forced home staying. In particular, the fear of the contagion, the quarantine imposed to the general population in Italy, and the negative experience associated with the COVID-19 pandemic affected food habits and promoted the development of maladaptive eating habits among obese patients waiting for bariatric surgery [9, 10].

Only one study [11], to date, investigated the effect of the COVID-19 pandemic and the lockdown on a waiting list cohort of 51 obese candidates for bariatric surgery; they found a small but statistically significant increase in BMI (42.7 to 43.2 kg/m^2^ in 2 months), although the severity of obesity measured through the Obesity Surgery Score did not show significant changes for BMI and obesity-related comorbidities.

The primary aim of this study was then to evaluate weight and food habit changes during COVID-19 outbreak since the psychological evaluation of patients eligible for bariatric procedure is crucial to assess their motivation, behavioral challenges, and emotional relationship with foods [12]. The secondary endpoint was to explore the psychological factors, arising during the pandemic, influencing weight and dietary variations.

## Materials and Methods

Obese patients waiting for bariatric procedures like as intragastric balloon position (IBP), gastric bypass (GBP), and sleeve gastrectomy (SG) were consecutively enrolled and retrospectively analyzed.

All patients underwent a dietary and psychiatric evaluation before the lockdown and virus spread period (according to International Guidelines for the evaluation of candidates for bariatric surgery) and were considered eligible for a bariatric procedure. All subjects who present at our outpatient service do sign a written informed consent (reviewed by our Ethical Committee) to have their clinical data potentially used for teaching and research purposes (provided that these data are anonymously treated).

Exclusion criteria were as follows: difficulty in contacting the patient by telephone and/or e-mail, inability in understanding the questions due to intellectual impairment, refusal to fill in the questionnaires, and poor Italian language knowledge.

After the end of the Italian lockdown (May 18), a survey was conducted by telephone interview including all obese patients waiting for the scheduled procedure. All contacted patients were briefed on the purpose of the study, and the ones that voluntary agreed to participate gave a verbal informed consent prior to the beginning of the survey. For this kind of study, our local ethical committee does not require a structured informed consent. The study was performed in accordance with the ethical standards laid down in the 1964 Declaration of Helsinki and its latter amendments.

The survey was composed of four different sections: the first one included anthropometric data (age, gender, weight, BMI before and after lockdown) and type of procedure; the second section was composed of the Hospital Anxiety and Depression Scale (HADS), a frequently used self-rating scale developed to assess psychological distress in non-psychiatric patients, together with the Zung Self-Rating Anxiety Scale (SAS) and the Zung Self-Rating Depression Scale (SDS), two validated instruments that patients filled in during the psychiatric evaluation before the pandemic; the third section investigated the presence of maladaptive eating behaviors (such as binge eating not satisfying criteria for binge eating disorder, night eating syndrome, emotional eating, gorging, snacking, grazing, sweet eating). In addition, in the fourth section, we designed a 4-item questionnaire investigating the personal feelings (boredom, fear of the virus, fear/distress due to the quarantine, and need to eat more to compensate for negative emotions) moved by the COVID-19 spread and lockdown.

### Statistical Analysis

Summary statistics of sociodemographic and clinical variables were expressed as mean and standard deviation, or counts and percentage, as appropriate. Comparisons between groups were made by the paired *t* test for continuous variables, and the chi-squared test for discrete variables.

We compared (a) weight and BMI before the lockdown and immediately after the end of the lockdown, (b) the proportion of individuals with maladaptive eating habits pre- versus post-lockdown, and (c) the SAS and SDS index scores together with the proportion of individuals with abnormal anxiety (SAS index score ≥ 45) and abnormal depression (SDS index score ≥ 45) before and at the end of the lockdown.

Finally, patients were divided in two groups according with their weight variation: weight increased and weight decreased group. Between groups, a comparison of sociodemographic, psychological, and clinical data was performed.

## Results

Ninety-five obese candidates for bariatric procedures were considered eligible after a dietary and psychiatric evaluation. Fifty-six accepted to be interviewed immediately after the end of the lockdown (*N* = 43 for gastric bypass, *N* = 1 for sleeve gastrectomy, and *N* = 12 for intragastric balloon position). Thirty-nine patients could not be reached neither by telephone nor by e-mails, and 10 refused or did not fill in the self-administered questionnaires for a total of 41.1% of excluded obese patients. Characteristics of individuals participating in the study are shown in Table 1.

**Table 1:** Comparison of sociodemographic and clinical characteristics before and after lockdown (*N* = 56)

*N* = 56	Before lockdown	After lockdown	*t*/*χ*^2^	df	*p*
Age, mean (± SD)	47.94 (9.25)	-			
Female gender, *N* (%)	38 (67.9)	-			
Weight (kg), mean (± SD)	118.17 (21.94)	118.32 (22.05)	− .176	55	.861
Height (m), mean (± SD)	1.67 (0.099)	-			
BMI (kg/m^2^), mean (± SD)	42.18 (5.72)	42.23 (5.79)	− .165	55	.869
Bariatric procedure, *N* (%)					
Gastric bypass	43 (76.8)				
Sleeve gastrectomy	1 (1.8)				
Intragastric balloon position	12 (21.4)				
Maladaptive eating habits (*N* = 54), *N* (%)					
Binge eating*	2 (3.7)	7 (13.0)	3.030	1	.082
Gorging	31 (57.4)	24 (44.4)	1.815	1	.178
Grazing/snacking	34 (63.0)	39 (72.2)	1.057	1	.304
Sweet eating	24 (44.4)	16 (29.6)	2.541	1	.111
Night eating	1 (1.9)	1 (1.9)	.000	1	1.000
Emotional eating	23 (42.6)	31 (57.4)	2.370	1	.124
HADS Anxiety score, mean (± SD)	-	5.70 (3.68)			
HADS Anxiety Abnormal (case) (score ≥ 11), *N* (%)	-	5 (8.9)			
HADS Depression score, mean (± SD)	-	4.75 (3.00)			
HADS Depression Abnormal (case) (score ≥ 11), *N* (%)	-	2 (3.6)			
SAS Anxiety index score, mean (± SD) **	*48.98 (11.54)*	*40.93 (8.81)*	*4.518*	*45*	*< .001*
SAS above normal range (score ≥ 45), N (%)**	*29 (63.0)*	*14 (30.4)*	*9.824*	*1*	*.002*
SDS Depression index score, mean (± SD)	45.52 (11.30)	42.76 (10.83)	1.908	45	.063
SDS above normal range (score ≥ 45), *N* (%)	23 (50.0)	17 (37.0)	1.592	1	.207

*BMI* = Body Mass Index; *HADS* = Hospital Anxiety and Depression Scale; *SAS Zung* = Self-rating Anxiety Scale; *SDS Zung* = Self-rating Depression Scale

*Criteria for bulimia nervosa or binge eating disorder not satisfied

**N = 46

During the lockdown, none of our patients were affected by the COVID-19 infection nor put in quarantine because of a family member positive for COVID-19; no significant changes in weight and BMI were detected during the 2.5 months of the lockdown: 118.17 ± 21.94 kg versus 118.32 ± 22.05 kg after the end of the lockdown (*p* = .861) and 42.18 ± 5.72 kg/m^2^ versus 42.23 ± 5.79 kg/m^2^ after the end of the lockdown (*p* = .869), respectively. Nevertheless, in 17.8% of cases (10/56), a change in obesity class was reported (Table 2), and among these patients, a substantial modification in bariatric procedures was planned (60%) (Table 3).

**Table 2:** Obesity class variation

*N* = 56	Before lockdown	After lockdown
Obesity class		
I (BMI 30–34,99)	4	5
II (BMI 35–39,99)	21	20
III (BMI > 40)	25	27
Super obese (BMI > 50)	6	3
Super super-obese (BMI > 60)	0	1

**Table 3:** Change in bariatric procedures

*N* = 10	Planned procedure changed	Planned procedure unchanged	*n* (%)	*p* value
Obesity class change				
Increased	1	3	10 (17.9%)	0.119
Decreased	5	1		
Total	6 (60%)	4 (40%)		

No statistically significant changes in the proportion of individuals showing maladaptive eating habits were observed during the lockdown (Table 1).

Interestingly, the lockdown and social distancing appeared to be associated with a reduced anxiety and depression scores in this obese population: a statistically significant reduction in the SAS anxiety index score was observed (48.98 ± 11.54 pre- versus 40.93 ± 8.81 post-lockdown, *p* < .001), together with a significant reduction in the proportion of individuals with an anxiety score above the normal range (63 to 30.4%, *p* = .002), while the reduction in the SDS index score and in the proportion with abnormal depression did not reach statistical significance (Table 1). At the HADS (administered at the end of the lockdown), only a minority of individuals scored above the cutoff for probable cases (Table 1), again indicating that avoiding social interactions for these severely obese individuals was associated with less anxiety and depression.

Thirty-two individuals gained weight during the lockdown (57.1%), while the remaining 24 were stable or showed a decrease in weight. The comparison between patients who gained weight and those who lost weight or remained stable is shown in Table 4.

**Table 4:** Comparison of sociodemographic and clinical characteristics between subjects with weigh increase versus weight decrease during the lockdown (*N* = 56)

*N* = 56	Weight increased *N =* 32	Weight decreased *N =* 24	*t*/*χ*^2^	df	*p*
Age, mean (± SD)	48.53 (9.51)	47.14 (9.02)	.554	54	.582
Female gender, *N* (%)	24 (75.0)	14 (58.3)	1.747	1	.186
Height (m), mean (± SD)	1.67 (0.09)	1.67 (0.11)	.206	54	.838
Weight pre-lockdown (kg), mean (± SD)	118.50 (21.72)	117.73 (22.70)	.128	54	.899
Weight post-lockdown (kg), mean (± SD)	122.29 (21.21)	113.01 (22.48)	1.580	54	.120
*Δ Weight, mean (± SD)*	*3.80 (4.14)*	*− 4.72 (5.10)*	*6.897*	*54*	*< .001*
BMI pre-lockdown (kg/m^2^), mean (± SD)	42.20 (5.70)	42.15 (5.87)	.026	54	.979
*BMI post-lockdown (kg/m*^*2*^*), mean (± SD)*	*43.59 (5.68)*	*40.41 (5.53)*	*2.094*	*54*	*.041*
*Δ BMI, mean (± SD)*	*1.39 (1.56)*	*− 1.74 (1.95)*	*6.690*	*54*	*< .001*
Bariatric procedure, *N* (%)			1.429	2	.489
Gastric bypass	23 (71.9)	20 (83.3)			
Sleeve gastrectomy	1 (3.1)	0 (0)			
Intragastric balloon position	8 (25.0)	4 (16.7)			
Maladaptive eating habits, *N (%)*					
Binge eating pre-lockdown*	2 (6.5)	0 (0.0)	1.541	1	.214
Binge eating post-lockdown*	6 (18.8)	1 (4.2)	2.667	1	.102
Gorging pre-lockdown	18 (58.1)	13 (56.5)	.013	1	.910
Gorging post-lockdown	14 (43.8)	10 (41.7)	.024	1	.876
Grazing/snacking pre-lockdown	22 (71.0)	12 (52.2)	2.000	1	.157
Grazing/snacking post-lockdown	26 (81.2)	15 (62.5)	2.459	1	.117
Sweet eating pre-lockdown	14 (42.2)	10 (43.5)	.015	1	.902
Sweet eating post-lockdown	9 (28.1)	9 (37.5)	.553	1	.457
Night eating pre-lockdown	0 (0)	1 (4.3)	1.373	1	.241
Night eating post-lockdown	1 (3.1)	0 (0)	.764	1	.382
Emotional eating pre-lockdown	14 (45.2)	9 (39.1)	.196	1	.658
Emotional eating post-lockdown	19 (59.4)	13 (54.2)	.152	1	.697
HADS Anxiety score, mean (± SD)	5.22 (3.47)	6.33 (3.92)	*−* 1.125	54	.266
HADS Anxiety Abnormal (case) (score ≥ 11), *N* (%)	3 (9.4)	2 (8.3)	.018	1	.892
HADS Depression score, mean (± SD)	5.00 (2.93)	4.42 (3.13)	.716	54	.477
HADS Depression Abnormal (case) (score ≥ 11), *N* (%)	1 (3.1)	1 (4.2)	.043	1	.835
SAS Anxiety index score pre-lockdown, mean (± SD)	47.16 (9.80)	50.22 (12.96)	*−* .971	50	.336
SAS Anxiety index score post-lockdown, mean (± SD)	40.31 (8.83)	41.41 (8.93)	*−* .428	46	.670
SDS Depression index score pre-lockdown, mean (± SD)	44.28 (9.99)	47.13 (11.92)	*−* .939	50	.352
SDS Depression index score post-lockdown, mean (± SD)	41.46 (9.86)	44.36 (11.81)	*−* .928	46	.358
During the lockdown, did you feel more…. than usual					
Bored	10 (31.2)	6 (25.0)	.263	1	.608
Fearful of contagion	15 (46.9)	13 (54.2)	.292	1	.589
*Distressed because of the lockdown*	*4 (12.5)*	*9 (37.5)*	*4.809*	*1*	*.028*
In need to eat more to compensate for negative emotions	7 (21.9)	4 (16.7)	.236	1	.627

*BMI *= Body Mass Index; *HADS* = Hospital Anxiety and Depression Scale; *SAS Zung* = Self-rating Anxiety Scale; *SDS Zung* = Self-rating Depression Scale

*Criteria for bulimia nervosa or binge eating disorder not satisfied

## Discussion

The primary aim of the study was to evaluate the impact of the COVID-19 pandemic and quarantine measures associated with it on weight (and BMI) and maladaptive eating behaviors among obese patients judged to be eligible for bariatric surgery and waiting for the surgical procedure.

All candidates usually undergo a thorough psychiatric examination before being considered eligible for bariatric surgery, by an in-person psychiatric interview using DSM-5 criteria, several self-administered rating scales and questionnaires (including SAS and SDS), and a specifically developed questionnaire investigating maladaptive eating behaviors.

We hypothesized that the fear of the contagion, the spread of the contagion in Italy during the first months of the year, and the associated measures (lockdown and quarantine) imposed by the government to face the pandemic, together with emotional reactions to these measures, would have been associated with a further increase in weight and BMI and with an increase in maladaptive eating habits.

In our cohort, the proportion of excluded patients (41.1%) is higher than the attrition rate after referral for bariatric surgery (22.9%, for example, in a study in Ontario) [13]; it is possible that some of the patient could have been affected by COVID-19 and/or been admitted to hospital or moved to other Italian regions less affected by the spread of the virus (our university town guests several students and teachers coming from other Italian regions), and this is a bias to bear in mind when examining our results.

Our hypothesis was disconfirmed: the mean weight before the lockdown was for the entire sample 118.17 ± 21.94 kg versus 118.32 ± 22.05 kg after the end of the lockdown (BMI pre 42.18 ± 5.72 kg/m^2^; BMI post 42.23 ± 5.79 kg/m^2^), without a statistically significant difference. The lockdown in Italy lasted from March 8 to May 18; obesity is a chronic condition with a multifactorial origin and with a very slow progressive increase in weight (and BMI) up to severe obesity. The relatively short duration of the lockdown (2 months approximately) as compared with the total length of time elapsed from beginning of weight gain up to severe obesity requiring surgical procedure did not translate into a further significant increase in weight/BMI. In addition, it is important to consider that the present study enrolled a selected cohort of motivated obese persons aiming to lose weight who received preoperative counseling with the aim of promoting compliance with the bariatric protocol. If the patients showed poor/absent motivation for losing weight and the willing to deeply adhere to the protocol at the preoperative psychiatric consultations, they would be excluded by the bariatric protocol, the operation and finally by this study.

Although the change in weight and BMI was not statistically significant, our study showed that a not negligible portion of sample modified its obesity class after the lockdown.

This purely theoretical result actually had a relevant clinical implication on the decision-making process. For all these patients, different and more appropriate bariatric procedures were proposed than those planned before the lockdown.

This study also filed to find significant differences in the rates of maladaptive eating habits; a large proportion of our sample showed maladaptive eating behaviors, presumably related to the increase in weight, but again, it appears that the lockdown and quarantine associated with the pandemic did not affect eating habits. The lack of weight gain and a non-significant variation in maladaptive eating habits are an indirect index of the efficacy and therefore of the essential need for a pre-operative selection process.

Interestingly, a significant decrease in anxiety (both the SAS anxiety index score and the proportion of individuals with abnormal anxiety) among severely obese individuals waiting for surgical procedure was found. Moreover, the proportion of individuals with abnormal scores on the HADS (both anxiety and depression) was very low. Contrary to the well-demonstrated negative effect of the pandemic and lockdown measures on psychological well-being of the general population [14–18], it appears that obese candidates waiting for surgical procedure do ameliorate during social isolation.

Concerning the effect of the pandemic and lockdown on weight in bariatric patients, our results are in line with the case-control study presented by Ruiz de Angulo and colleagues [19], who found no effect of the lockdown on weight loss after vertical gastrectomy, but in contrast with the small but significant increase in BMI associated with the 2-month lockdown in a recent study among 51 obese candidates for bariatric surgery [11].

Moreover, our results of a decrease in anxiety with no changes in depression scores during the lockdown are in contrast with other data reported in the limited literature available, reporting a significant increase of both anxiety and depression in patients included in a bariatric surgery program [9, 20, 21] and patients with obesity [22, 23]). It is to be considered that in these studies, a mixed cohort of patients waiting for different types of procedures were analyzed and the largest sample was represented by post-surgical patients. Our hypothesis is that the improvement in anxiety may be related to the severe body dissatisfaction/uneasiness experienced by severely obese individuals, often associated with the fear of confronting with others (social phobic features) and be negatively judged by them, leading to avoidance of social interactions situations; it is then possible that the “positive” effect of being obliged to avoid social interactions during the lockdown may have been greater than the fear of being infected by the COVID-19, leading to a significant reduction in anxiety (while depression scores did not change).

Several limitations need to be acknowledged. Most of them are common to other papers present in literature [11]. Firstly, we analyzed an obese population waiting for different bariatric procedures, with different baseline data and expectations about the scheduled treatment.

Additionally, as reported above, 39 subjects could not be reached neither by telephone nor by e-mails. We chose to use both the telephone interview and an online survey to be sure of reaching potential participants (through which participants filled in the questionnaires). This modality was chosen in order to reduce the discomfort/distress eventually associated with an e-mail invitation. None of our potential participants explicitly reported any discomfort, when asked to answer the online survey. However, ten individuals refused or did not fill in the self-administered questionnaires, and this limits the generalizability of our results. Finally, the absence of a control cohort of patients waiting for other health treatments that possibly could have helped to understand if our results on the effect of lockdown due to COVID-19 pandemic were specific for obese patients or could be extended to general population [11].

A strength of this study was in the use of specific questionnaires exploring anxiety and depression filled in by the same patients both before and after the lockdown period. In addition, few studies in literature exploring psychological distress and weight changes in the preoperative period are available, and this paper tries to demonstrate that avoiding social interactions for these severely obese patients is associated with less anxiety and depression.

In conclusion, while facing a second wave of the pandemic all over the world, together with new lockdown measures, our study showed no effect on weight and BMI nor on rates of maladaptive eating habits associated with quarantine/social isolation among severely obese individuals waiting for the bariatric surgery. Although the weight variation was non-significant, a considerable proportion of patients changed the initial obesity class. Thus, surgeons and patients at the end of the lockdown should take into account a possible change of bariatric procedure compared with the planned one. Interestingly, a reduction in anxiety and depression scores was reported probably associated with the limited social interactions due to the lockdown and social distancing.

## Declarations

The informed consent was obtained from all individual participants included in the study, under the institutional review board policies of hospital administration. There are no relationships with industry.

## References

[1] World Health Organization, WHO. WHO Director-General’s opening remarks at the media briefing on COVID-19 - 11 March 2020. Available online: https://www.who.int/dg/speeches/detail/who-director-general-s-opening-remarks-at-the-media-briefing-on-covid-19%2D%2D-11-march-2020 (accessed on 9 May 2020)

[2] Li X, Wang W, Zhao X, et al. Transmission dynamics and evolutionary history of 2019-nCoV. J Med Virol 2020b. 10.1002/jmv.25701 PMC7166881 32027035

[3] Zhu N, Zhang D, Wang W (2019). A novel coronavirus from patients with pneumonia in China, 2019. N Engl J Med.

[4] Hussain A, Mahawar K, El-Hasani S. The impact of COVID-19 pandemic on obesity and bariatric surgery. Obes Surg. 2020:1–2. 10.1007/s11695-020-04637-7. 10.1007/s11695-020-04637-7 PMC7211046 32388706

[5] Søreide K, Hallet J, Matthews JB, et al. Immediate and long-term impact of the COVID-19 pandemic on delivery of surgical services. Br J Surg. 2020. 10.1002/bjs.11670. 10.1002/bjs.11670 PMC7267363 32350857

[6] Yang W, Wang C, Shikora S, et al. Recommendations for metabolic and bariatric surgery during the COVID-19 pandemic from IFSO. Obes. Surg. 2020:1–3. 10.1007/s11695-020-04578-1 PMC7155392 32291701

[7] Taube-Schiff M, Van Exan J, Tanaka R, Wnuk S, Hawa R, Sockalingam S (2015). Attachment style and emotional eating in bariatric surgery candidates: the mediating role of difficul- ties in emotion regulation. Eat Behav.

[8] Shakory S, Van Exan J, Mills JS, Sockalingam S, Keating L, Taube-Schiff M (2015). Binge eating in bariatric surgery candidates: the role of insecure attachment and emotion regulation. Appetite.

[9] Sockalingam S, Leung SE, Cassin SE (2020). The impact of coronavirus disease 2019 on bariatric surgery: redefining psychosocial care. Obesity (Silver Spring)..

[10] Bhasker AG, Greve JW (2020). Are patients suffering from severe obesity getting a raw deal during COVID-19 pandemic?. Obes Surg (Silver Spring)..

[11] Beisani M, Vilallonga R, Petrola C, et al. Effects of COVID-19 lockdown on a bariatric surgery waiting list cohort and its influence in surgical risk perception. Langenbeck’s Arch Surg. 2020; 10.1007/s00423-020-02040-5. 10.1007/s00423-020-02040-5 PMC7690848 33244718

[12] Snyder AG (2009). Psychological assessment of the patient undergoing bariatric surgery. Ochsner J.

[13] Doumouras AG, Lee Y, Babe G, Gmora S, Tarride JE, Hong D, Anvari M (2020). The hidden cost of an extensive preoperative work-up: predictors of attrition after referral for bariatric surgery in a universal healthcare system. Surg Endosc..

[14] Brooks SK, Webster RK, Smith LE, Woodland L, Wessely S, Greenberg N, Rubin GJ (2020). The psychological impact of quarantine and how to reduce it: rapid review of the evidence. Lancet.

[15] Lima CKT, Carvalho PMM, Lima IAAS, Nunes JVAO, Saraiva JS, de Souza RI (2020). The emotional impact of Coronavirus 2019-nCoV (new Coronavirus disease). Psychiatry Res.

[16] Hanna F, Barbui C, Dua T, Lora A, van Regteren AM, Saxena S (2018). Global mental health: how are we doing?. World Psychiatry.

[17] Venkatesh A, Edirappuli S (2020). Social distancing in covid-19: what are the mental health implications?. BMJ.

[18] Fiorillo A, Sampogna G, Giallonardo V, Del Vecchio V, Luciano M, Albert U, Carmassi C, Carrà G, Cirulli F, Dell’Osso B, Nanni MG, Pompili M, Sani G, Tortorella A, Volpe U (2020). Effects of the lockdown on the mental health of the general population during the COVID-19 pandemic in Italy: results from the COMET collaborative network. Eur Psychiatry.

[19] Ruiz de Angulo D, Balaguer Román A, Munitiz Ruiz V, Gil Vázquez PJ, Ruiz Merino G, Ortiz Escandell MÁ, Martínez de Haro LF, Parrilla Paricio P (2020). Influence of the lockdown due to COVID-19 on ponderal results during the first year after vertical gastrectomy. Cir Esp.

[20] Sisto A, Vicinanza F, Tuccinardi D, Watanabe M, Gallo IF, D’Alessio R, Manfrini S, Quintiliani L (2020). The psychological impact of COVID-19 pandemic on patients included in a bariatric surgery program. Eat Weight Disord..

[21] Reynolds DL, Garay JR, Deamond SL, et al. Understanding, compliance and psychological impact of the SARS quarantine experience. Epidemiol Infect. 2008;136(7) 10.1017/S0950268807009156. 10.1017/S0950268807009156 PMC2870884 17662167

[22] Almandoz JP, Xie L, Schellinger JN, Mathew MS, Gazda C, Ofori A, Kukreja S, Messiah SE (2020). Impact of COVID-19 stay-at-home orders on weight-related behaviours among patients with obesity. Clin Obes..

[23] Simon GE, von Korff M, Saunders K, Miglioretti DL, Crane PK, van Belle G, Kessler RC (2006). Association between obesity and psychiatric disorders in the US adult population. Arch Gen Psychiatry..

